# The effect of negative pressures on the superconductivity of amorphous and crystalline bismuth

**DOI:** 10.1038/s41598-022-22261-6

**Published:** 2022-11-11

**Authors:** Flor B. Quiroga, David Hinojosa-Romero, Alexander Valladares, Renela M. Valladares, Isaías Rodríguez, Ariel A. Valladares

**Affiliations:** 1 Instituto de Investigaciones en Materiales, Universidad Nacional Autónoma de México, Apartado Postal 70-360, Ciudad Universitaria, 04510 CDMX, México; 2 Facultad de Ciencias, Universidad Nacional Autónoma de México, Apartado Postal 70-542, Ciudad Universitaria, 04510 CDMX, México

**Keywords:** Electronic structure, Structure of solids and liquids, Superconducting properties and materials, Density functional theory

## Abstract

Materials may behave in non-expected ways when subject to unexpected conditions. For example, when Bi was turned into an amorphous phase (a-Bi) unexpectedly it became a superconductor at temperatures below 10 K. Using the superconductivity of the amorphous phase we provided an explanation as to why crystalline bismuth (c-Bi) had not been found to superconduct, and even predicted an upper limit for its superconducting transition temperature *T*_*c*_. This was experimentally corroborated within the following year. We now decided to investigate what happens to the crystalline, Wyckoff structure, and amorphous Bi when pressures below the atmospheric are applied. Here it is shown that, within the BCS approach, under expansion the Wyckoff c-Bi increases its superconducting transition temperature minimally, whereas the amorphous phase decreases its *T*_*c*_. The electron densities of states (eDoS), the vibrational densities of states (vDoS) and the Debye temperatures (*θ*_*D*_) are calculated to perform this qualitative evaluation. Expansion can be obtained in the laboratory by chemically etching Bi-based alloys, for example, a process also known as dealloying.

## Introduction

The quest for a room temperature superconductor has permeated the field for decades. Patents and claims have come and gone^1,2^ and with the advent of laboratory equipment that produces very high pressures for very short periods of time the quest has increased. Recently, a (controversial) claim, portrayed in a paper on carbonaceous sulfur hydride^3^ that supposedly is a room temperature superconductor under very high pressures, provoked an optimism that gave hope to accomplish this quest for more accessible pressures. However, the high pressures required thermalize the original optimism since the difficulties involved in the process are considerable, and it does not seem possible to develop a room temperature superconductor at reasonable pressures yet. These experimental results were questioned by some researchers who did not give credit to the claim^4^ (see Ref.^5^ also), disbelieve that has not been fully clarified. But, going high in pressure is the only resource to investigate high *T*_*c*_ superconductivity? In principle other alternatives should be explored. Faced with the above controversy and searching for other alternatives it seemed natural “to look the other way” and start studying the effect on the electronic structure and vibrational properties of materials when pressures below atmospheric are applied, (“negative” pressures). Since one of the superconductors better investigated is bismuth in its solid phases, both at atmospheric pressure and also at high pressures, it was decided to undertake its study under pressures below the atmospheric, although being a heavy element no record-breaking changes in *T*_*c*_ were expected. It happens that when a new field of research is developed other lines are essentially forgotten and that is why, in this work, we are looking the other way to search for interesting properties or results with the hope of catalyzing developments hitherto minimized.

Bismuth is an interesting material since it is one of the few elements that maintains some properties, like superconductivity, under varied circumstances. Bismuth was first found to be a superconductor when in the amorphous phase at ambient pressure^6,7^, with a superconducting transition temperature of ∼ 6 K. Then, when the Wyckoff crystalline phase (a rhombohedral structure with the *R-3 m* space group, at room temperature and atmospheric pressure) was subjected to positive pressures it changed crystalline structures but maintained the superconducting properties for most of the new topologies (see Refs.^8,9^, and references contained therein). However, the possible superconductivity of c-Bi in the Wyckoff structure had not been found so we decided to study it based on the experimental results for the amorphous phase. We predicted, using a simple BCS approach, that although the crystalline Wyckoff structure had not been found to super conduct, it should, for temperatures below 1.3 mK^10^. A year after our prediction, experimentalists proved us correct and they found that the Wyckoff structure super conducts at 0.53 mK at ambient pressure^11^.

To predict the crystalline superconducting transition temperature, we calculated the electronic density of states, eDoS, the vibrational density of states, vDoS, and the Debye temperature, *θ*_*D*_, and utilizing the experimentally known *T*_*c*_ for amorphous bismuth at ambient pressure, the possible superconductivity of the Wyckoff structure was studied. The values for *θ*_*D*_ were 100 K for the amorphous and 129 K for the crystalline, whereas the eDoS for the amorphous was of the order of 4 times larger than the value for the crystalline at the Fermi level. The contribution of Mata-Pinzón et al*.*^10^ resides in finding a process that led to an amorphous structure of bismuth with pair distribution functions in agreement with the lone experiment by Fujime^12^. Then the calculation of some physical properties of this structure was considered realistic; so, by relating the unknown superconducting transition temperature of the crystalline phase to known parameters of the amorphous phase (assuming that the electron–phonon Cooper interaction did not change during the phase change) an estimated result of 1.3 mK was obtained. This procedure will be used here and will be better discussed later in this work.

So far, we have ventured to estimate superconducting temperatures for bismuth under various conditions. For the crystalline phase, that had not been found to super conduct, we succeeded; however, our other predictions await verification^9,13^. So, with this succinct background it was evident that bismuth was the ideal “guinea pig” to test the effect of negative pressures and the search was undertaken to see if the superconductivity predicted for the crystalline and experimentally determined for the amorphous could be altered by the expansion. This is the origin of this work.

The changes that take place in the eDoS and the vDoS for the Wyckoff bismuth when expanded 5%, 10%, 15% its ambient volume, are reported. We shall then consider the amorphous phases of these expanded structures to investigate how the electronic and vibrational properties change. To search for variations of superconductivity, we have to apply the approach stated in Ref.^8^, to study the effect of the expansion on the electronic and vibrational properties of the material, and from there calculate *T*_*c*_.

Studies on the modifications that occur under expansion of other solids have been reported in the literature like the work of Moruzzi and Marcus whom in 1989 investigated the electronic changes that occur in solid palladium under negative pressures^14^. They found that Pd may become magnetic when expanded. Thirty years later, in 2019, we investigated the electronic properties of amorphous^15^ and amorphous-porous palladium [to be published]. In both cases we found that when the stable crystalline structure of Pd is altered, magnetism appears. The field of solids under expansion is not as well researched due to the difficulties of experimentally creating an expanded sample. However, developments, like the chemical etching of alloys, seem promising to foster an incursion in this field since the removal of one of the atomic species by chemical methods diminishes the density of the material, creating de facto an expansion of the sample with one type of atom, despite other changes that may occur. Also, from the computational point of view this does not represent an insurmountable problem as we shall present in what follows.

In the following section we deal with the methodology that we used. Next, we present the results of our investigation, to go sequentially to analyze such results and infer possible consequences of our predictions. Finally, we elaborate on the conclusions as to what the implications are and to further our speculations for future work.

## Methodology. The structure

When dealing with materials that have not been experimentally realized one has to very careful not to create science fiction. In this work we assume that the crystalline Wyckoff structure, stable at room temperature and at atmospheric pressure, remains the same when expanded up to 15%, which might not be the case. However, when amorphized, this handicap disappears and no memory of the original structure remains which then serves for comparison purposes.

**Figure 1:**
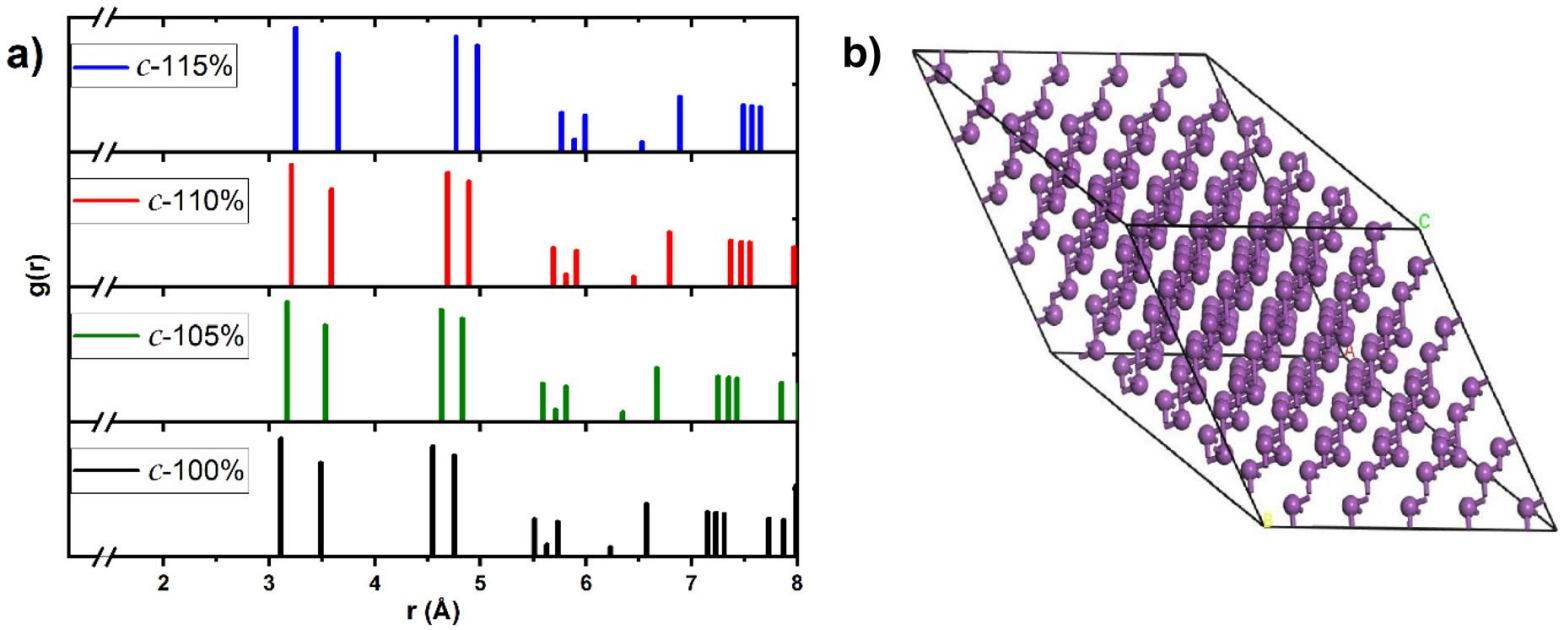
(**a**) PDFs for the crystalline supercell structures. The positions of the peaks undergo a displacement consistent with the expansion forced on the structures. (**b**) 250-atom supercell of the Wyckoff structure where the bilayers can be seen. Linked atoms are nearest neighbors. (Fig. 1b was obtained using the Dassault Systèmes BIOVIA Materials Studio software, version 2016-1^17^, https://www.3ds.com/products-services/biovia/products/molecular-modeling-simulation/biovia-materials-studio/).

**Figure 2:**
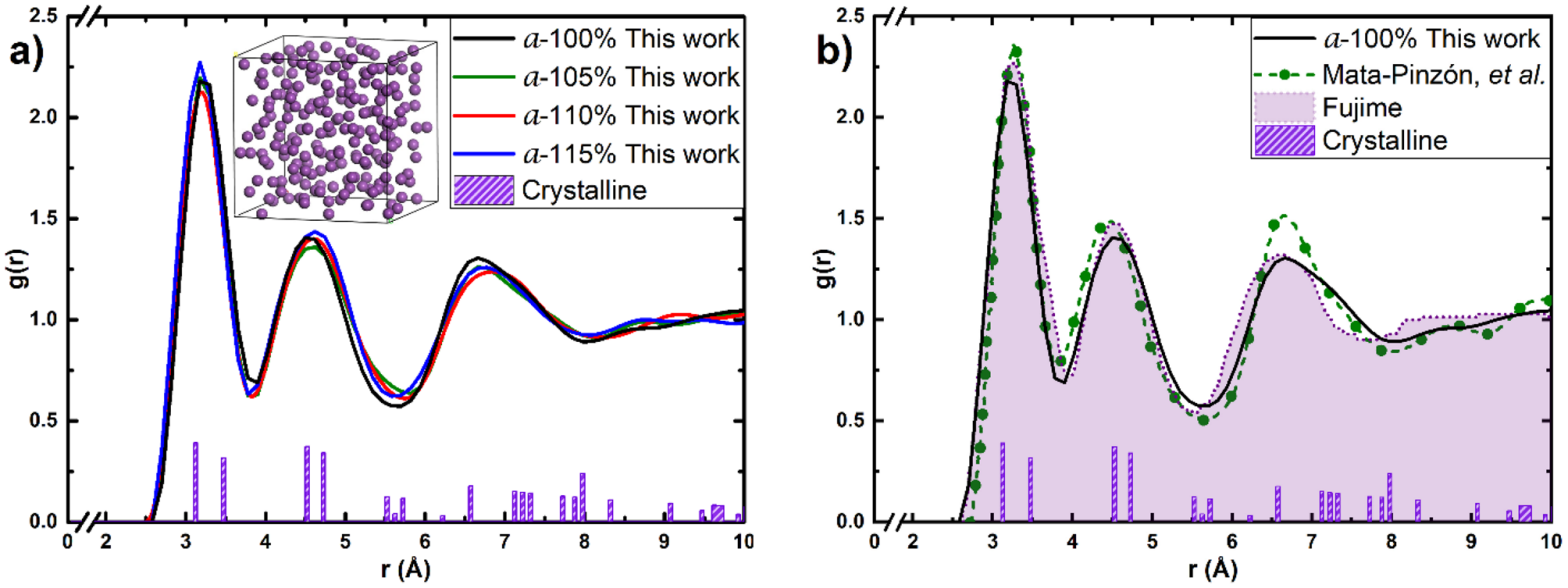
(**a**) PDFs for the amorphous supercells generated from the non-expanded and the expanded structures, at the end of the GO process. The 216-atom a-Bi 100% supercell is shown in the inset. (**b**) Comparison with the simulational results of Mata-Pinzón et al.^10^ and the experiment reported by Fujime^12^. The crystalline non-expanded PDFs are also included for comparison.

Superlattices for the crystalline and for the amorphous samples were constructed. For the crystalline structures the superlattices contained 250 atoms as a result of multiplying the Wyckoff rhombohedral unit cell 5 × 5 × 5 times. For the amorphous the supercell contained 216 atoms and were obtained by multiplying the diamond-like cubic unit cell 3 × 3 × 3 times. The diamond-like structure is an initially unstable structure to propitiate the evolution into a disordered topology, maintaining the correct density for the non-expanded structure (9.81 g/cm^3^). After that we expanded both superlattices 5, 10 and 15% times, properly scaling the interatomic distances, to investigate the evolution of the electron densities of states and the vibrational densities of states. In Table 1 we specify the lattice parameters and densities for all our samples. For the amorphous we ran our *undermelt-quench* procedure^16^ to generate the corresponding samples. Figures 1 and 2 represent the Pair Distribution Functions (PDFs) for the crystalline and for the amorphous, respectively. In Fig. 2 we have included the experimental results by Fujime^12^, and the agreement with our non-expanded amorphous sample is good. Also, in Fig. 2 we present the PDF for the non-expanded Wyckoff *crystalline* structure, for comparison.

**Table 1:** Parameters of our crystalline (c-) and amorphous (a-) samples.

System	Lattice parameter	Density
	(Å)	(g cm^−3^)
c-100%	23.73	9.81
a-100%	19.70	
c-105%	24.12	9.34
a-105%	20.02	
c-110%	24.50	8.91
a-110%	20.33	
c-115%	24.86	8.53
a-115%	20.64

For the sake of completeness and self-containment the description of the undermelt-quench procedure^16^ is briefly reproduced. The procedure starts with an *unstable* crystalline structure, with the correct density, to help the amorphizing procedure; usually for the metallic systems studied, we chose a diamond-like structure to initiate the process. Then a Molecular Dynamics (MD) is performed starting from 300 K linearly heating the sample to just below the melting temperature (*undermelt*). Next we linearly cool the sample down to close to absolute zero (*quench*). At the end of this MD process the structure is essentially disordered, but evidently unstable. To favor the final locally-stable structure a Geometry Optimization (GO) procedure is carried out which generates amorphous structures that agree well with experimental results when available; that is why we consider the process adequate to predict structures that are presently not known, with their corresponding PDFs^15^. Once the GO process is completed the eDoS and vDoS are calculated for all the structures generated. It should be clear at this point that the amorphous supercell generated has periodic boundary conditions but we claim that it represents the material for distances comparable to the dimensions of the supercell. The periodicity is spurious but helps to calculate the physics of properties that do not extend beyond the size of the superlattice.

For all calculations, we employed a Density Functional Theory (DFT) approach as implemented in the DMol3 code^18^, part of the Materials Studio suite^17^. The electronic treatment consisted in a double numerical basis set with d-function polarization (dnd) and a 6.0 Å real-space cutoff. We used the Vosko-Wilk-Nusair (VWN) functional^19^ within the Local Density Approximation (LDA) for the computation of the exchange-correlation energy and DFT Semi-core PseudoPotentials (dspp) for the core treatment^20^. All calculations were spin-unrestricted with Self Consistent Field (SCF) density convergence of 1 × 10^–6^ using a thermal smearing of 0.136 eV. It should be mentioned that the present results were obtained with the Materials Studio 2016 version, whereas the results of Ref.^10^ were obtained with an older version of the Materials Studio Modeling suite, the 6.0.

For the construction of the amorphous samples, the MD process uses a Nosé-Hoover^21,22^ NVT thermostat with a Nosé Q-ratio of 0.5 and a time step of 19.4 fs. The undermelt-quench heating ramp started at 300 K and reached 534 K in 100 steps of 2.34 K per step, followed by a cooling ramp of 2.34 K per step also, down to 5.16 K. For the GO procedure the convergence was sought by using the following thresholds: 2.7 × 10^–4^ eV, 2 × 10^–3^ eV Å^−1^ and 5 × 10^–3^ Å for energy, maximum force and maximum displacement, respectively.

The single-point energy and eDoS calculations for each geometrical structure were conducted for a mesh of *k*-points determined by a 3 × 3 × 3 cross-linked structure in the first Brillouin zone. To represent the bulk material, a 0.12 eV gaussian broadening was applied to the eigenvalues calculated.

To obtain the vibrational frequencies, a single-point energy calculation at the *Γ*-point of the Brillouin zone was conducted to compute the mass-weighted Hessian matrix through finite differences of the energy first derivatives with a step size of 0.005 Å. The vDoS, *F*(*ω*), for each system was then drew using a 3-point Fast Fourier Transform of the frequency distributions with a bin width of 0.25 meV.

A word on nomenclature, the non-expanded samples are referred to as the 100% structures, whereas the samples expanded *x*% are referred to as (10*x*)%, or to (1*x*)%, when x is a two-digit number.

## Results and analysis

### The electron density of states at the Fermi level

**Figure 3:**
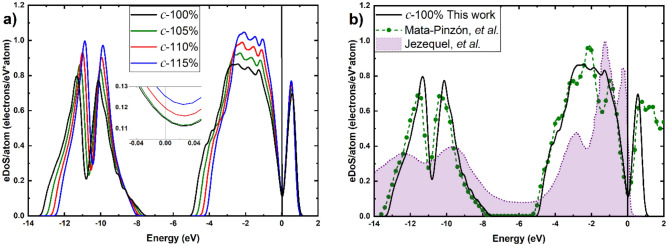
(**a**) eDoS per atom for the non-expanded and the expanded crystalline supercells of this work, obtained with a mesh of 3 × 3 × 3 *k*-points. The inset shows the behavior in the vicinity of the Fermi energy. (**b**) Our eDoS for 100% c-Bi compared to the simulations of Ref.^10^ and to the experiments of Jezequel et al.^23^.

**Figure 4:**
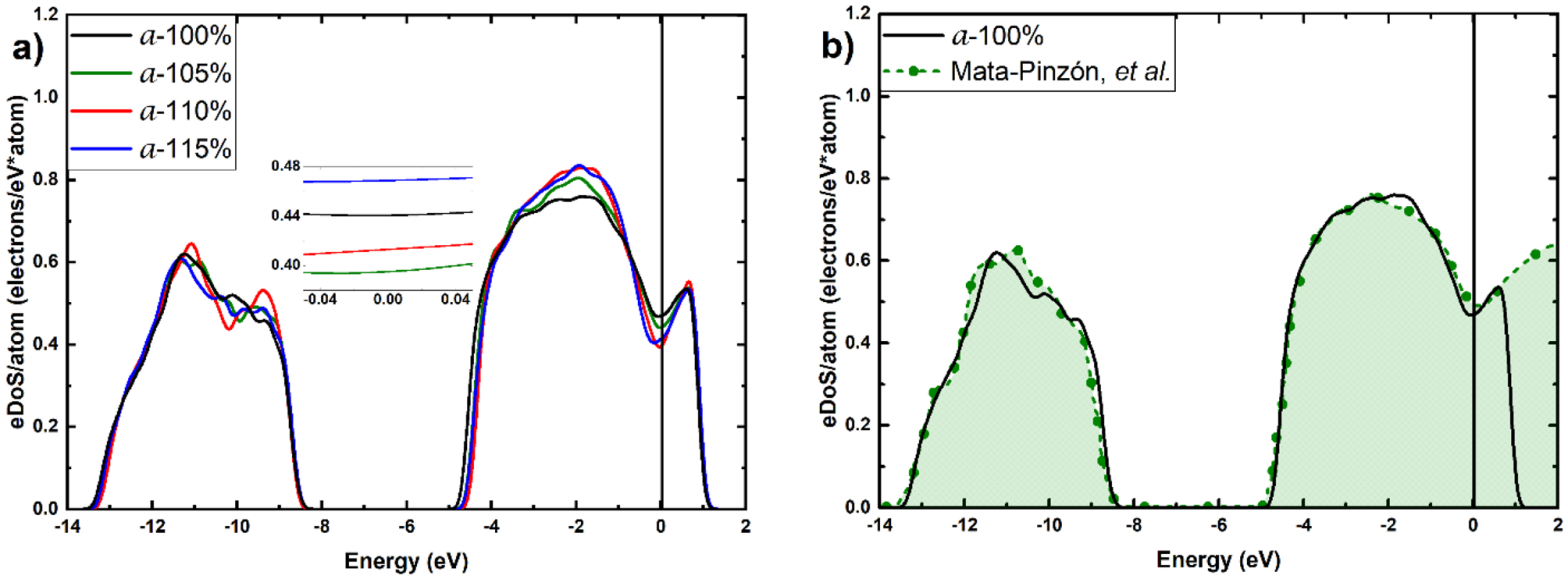
(**a**) eDoS per atom for the non-expanded and the expanded amorphous supercells of this work, obtained with a mesh of 3 × 3 × 3 *k*-points. The inset shows the behavior in the vicinity of the Fermi energy. (**b**) Our eDoS for 100% a-Bi compared to the simulations Ref.^10^

The calculated eDoS are represented in Fig. 3a,b obtained for a 3 × 3 × 3 mesh of *k-*points for the crystalline structures in the first Brillouin zone, and in Fig. 4 for the amorphous samples also obtained for a mesh of 3 × 3 × 3 k-points, after the GO processes. Since the runs for the crystalline are done on supercells with 250 atoms, whereas for the amorphous we considered supercells with 216 atoms, the eDoS are reported per atom to normalize our results and to compare them readily.

Since our purpose is to investigate the behavior of the eDoS curves in the vicinity of the Fermi energy, Figs. 3,4 show this behavior in the insets for the structures considered. Since the parameters used for these calculations are the same for all samples, the results are directly compared. In this manner we can concentrate on visualizing, contrasting and analyzing all our results, for both non-expanded and expanded, crystalline and amorphous, structures. An expected result is that the amorphous samples have a larger eDoS than the crystalline ones in all cases studied here. This was expected because that was the origin of our proposal to predict that the Wyckoff structure should super conduct below 1.3 mK^10^. However, an unexpected result is to see that the eDoS for the amorphous diminishes with the expansion, while the opposite occurs for the crystalline ones. This could be due to the fact that as the amorphous expands, the tendency is to become more crystalline-like since the appearance of pores as a result of the increase in volume fosters the formation of small regions where the structure is not condition by the amorphicity and tends to crystallize. The opposite happens with the expanded crystalline cells as they rearrange to cope with this expansion. It may be possible that eventually, the tendencies will approach one another and become indistinguishable. Further calculations are needed to discern this supposition.

### The vibrational density of states

The vibrational density of states of amorphous materials became an interesting subject when during the second half of the last century, Zeller and Pohl^24^ discovered in 1971 that the specific heats of amorphous solids differ from their crystalline counterparts at low temperatures. This implies that the Debye approximation at low temperatures does not hold, and the *ω*^*2*^ dependence gives way to a different behavior for the vibrational density of states. Such a finding provoked an avalanche of models since this result became a challenge for theorists, and a number of supposed explanations were proposed. These models shall not be discussed here, what we want to discuss is the effect of the amorphicity in the vDoS and its consequences in the probable superconductivity of bismuth.

**Figure 5:**
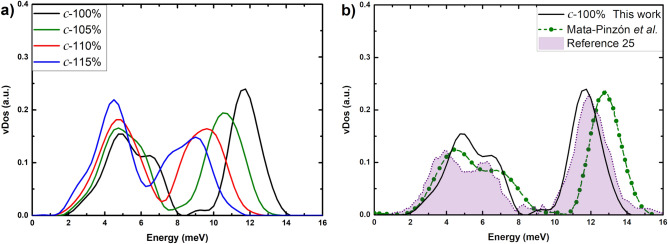
vDoS for (**a**) the non-expanded and expanded crystalline structures, and for (**b**) 100% c-Bi compared to the simulations of Ref.^10^ and to the results  of reference^ 25^.

**Figure 6:**
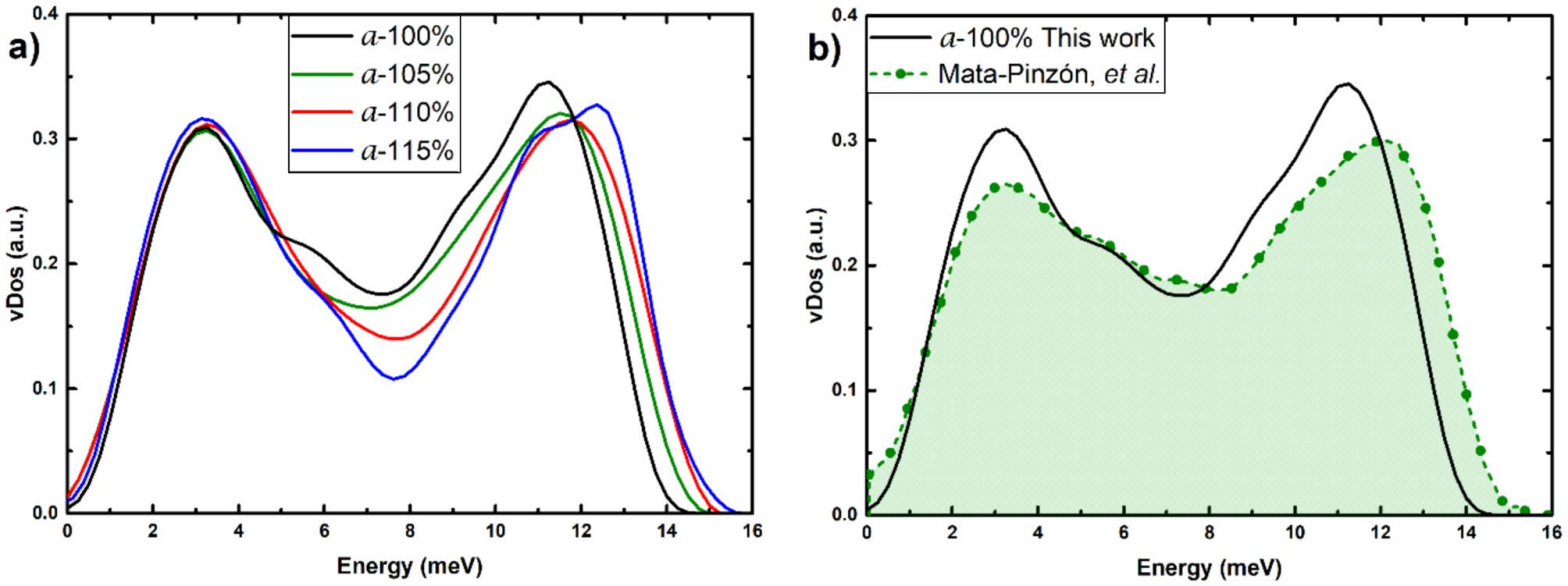
vDoS for (**a**) the non-expanded and expanded amorphous structures, and for (**b**) 100% a-Bi compared to the simulations of Ref.^10^

In Figs. 5,6 the results of the vDoS calculations for the crystalline and the amorphous samples, as a function of the expansion, are plotted. One can see the interesting changes that take place in the vibrational spectrum of each cell. The 100% c-Bi sample displays a gap in the spectrum that indicates a layered atomic structure, as is well known. As the expansion grows, this layered structure tends to disappear until for the sample 115% c-Bi, a pseudo-gap appears. The whole spectrum moves towards lower energies as the “spring constant” of the force between atoms weakens due to the expansion. Interestingly enough the lowest energies of the vDoS do not displace much as opposed to the higher energy modes. For the amorphous, expansion does not seem to affect them much; they start with a pseudo-gap that remains through the expanding process. The low frequency modes (soft modes) do not vary much, similarly to what occurs in the crystalline case. The high frequency modes, however, do experience a “hardening” of the frequencies, unlike the crystalline.

Soft phonon modes have been invoked as a strong characteristic to influence the superconductivity of materials. So the fact that they increase for the crystalline as a function of the expansion may indicate an enhancement of the *T*_*c*_ for the material. Also, the decrease of these modes for the amorphous may indicate a decrement in *T*_*c*_. These speculations will be quantified in the next section by taking into account the contributions of the eDoS and the Cooper pairing potential *V* to the superconducting transition temperatures of our supercells, both expanded and non-expanded.

### Possible superconductivity a la BCS

What would be the effect of the above-mentioned results on the superconductivity of the cells studied? If we think in terms of the BCS theory, the superconducting transition temperatures *T*_*c*_ is given by.

$${\text{T}}_{{\text{c}}} \, = \,1.13\theta_{D} \,exp \, \left( { - 1/N\left( {E_{F} } \right)V} \right),$$

where *N(E*_*F*_*)* is the eDoS at the Fermi level, *θ*_*D*_ is obtained from the vDoS using Grimvall’s expression (see below), and *V* is the strength of Cooper ‘s phonon mediated electron–electron attractive potential. Since there are changes in these quantities, one should expect changes in the value of the superconducting transition temperature *T*_*c*_. To estimate these changes, we followed the procedure first proposed by Mata-Pinzón, et al*.*^10^. This procedure, which is outlined next, considers the electron–phonon interaction, *V*, to be nearly constant whether bismuth is in its crystalline or in its amorphous structure, and requires the computation of *N(E*_*F*_*) a*nd *θ*_*D*_* (*= *ħω*_*D*_*/k*_*B*_), for all the samples.

*N(E*_*F*_*)* is readily obtained through the calculated eDoS. *θ*_*D*_ is obtained through the equation ^26^:

$${\Theta }_{D}=\frac{\hslash }{{k}_{B}}{\omega }_{D}=\frac{\hslash }{{k}_{B}}\mathrm{exp}\left[\frac{1}{3}+\frac{{\int }_{0}^{{\omega }_{\mathrm{max}}}\mathrm{ln}(\omega )F(\omega )d\omega }{{\int }_{0}^{{\omega }_{\mathrm{max}}}F\left(\omega \right)d\omega }\right]$$

where *k*_*B*_ is the Boltzmann constant, *F(ω)* is the vDoS for a given structure and *ω*_*max*_ is the maximum frequency of the calculated and smoothed *F(ω)*. The value of *ω*_*D*_ given by the equation above is, according to Grimvall, an upper limit to the real value of the Debye frequency, and this should be kept in mind when comparing with experiment.

Let *N(E*_*F*_*)*^*s,z*^ be the eDoS at the Fermi level for the *z* sample (*z* = 100, 105, 110 and 115%) corresponding to the *s* structure (*s* is *a* for the amorphous and *c* for the crystalline). In particular, let *N(E*_*F*_*)*^a,100%^ be the eDoS at the Fermi level for the non-expanded amorphous sample; *N(E*_*F*_*)*^a,100%^ and *N(E*_*F*_*)*^*s,z*^ are related through the proportionality constant *α*_*s,z*_ as:

$$N{\left({E}_{F}\right)}^{a,100\%}= {\alpha }_{s,z}N{\left({E}_{F}\right)}^{s,z}.$$

In the same manner, let *θ*_*D*_^*s,z*^ be the Debye temperature for the *s* structure of the *z* sample. In particular, *θ*_*D*_^a,100%^ is the Debye temperature for the non-expanded amorphous sample; *θ*_*D*_^a,100%^ and *θ*_*D*_^*s,z*^ are related through the proportionality constant *β*_*s,z*_ as:

$${\beta }_{s,z}{{\Theta }_{D}}^{a,100\%}= {{\Theta }_{D}}^{s,z}.$$

Following Mata-Pinzón et al., the superconducting transition temperature for the *s* structure of the *z* sample, *T*_*C*_^*s*,*z*^, can be estimated with the aid of a reference measured temperature, *T*_*C*_^a,100%^, which in this case corresponds to the non-expanded amorphous supercell:

$${{T}_{c}}^{s,z}={\beta }_{s,z}{\left(1.13\, {{\Theta }_{D}}^{a,100\%}\right)}^{1-{\alpha }_{s,z}}{\left({{T}_{c}}^{a,100\%}\right)}^{{\alpha }_{s,z}}$$

**Figure 7:**
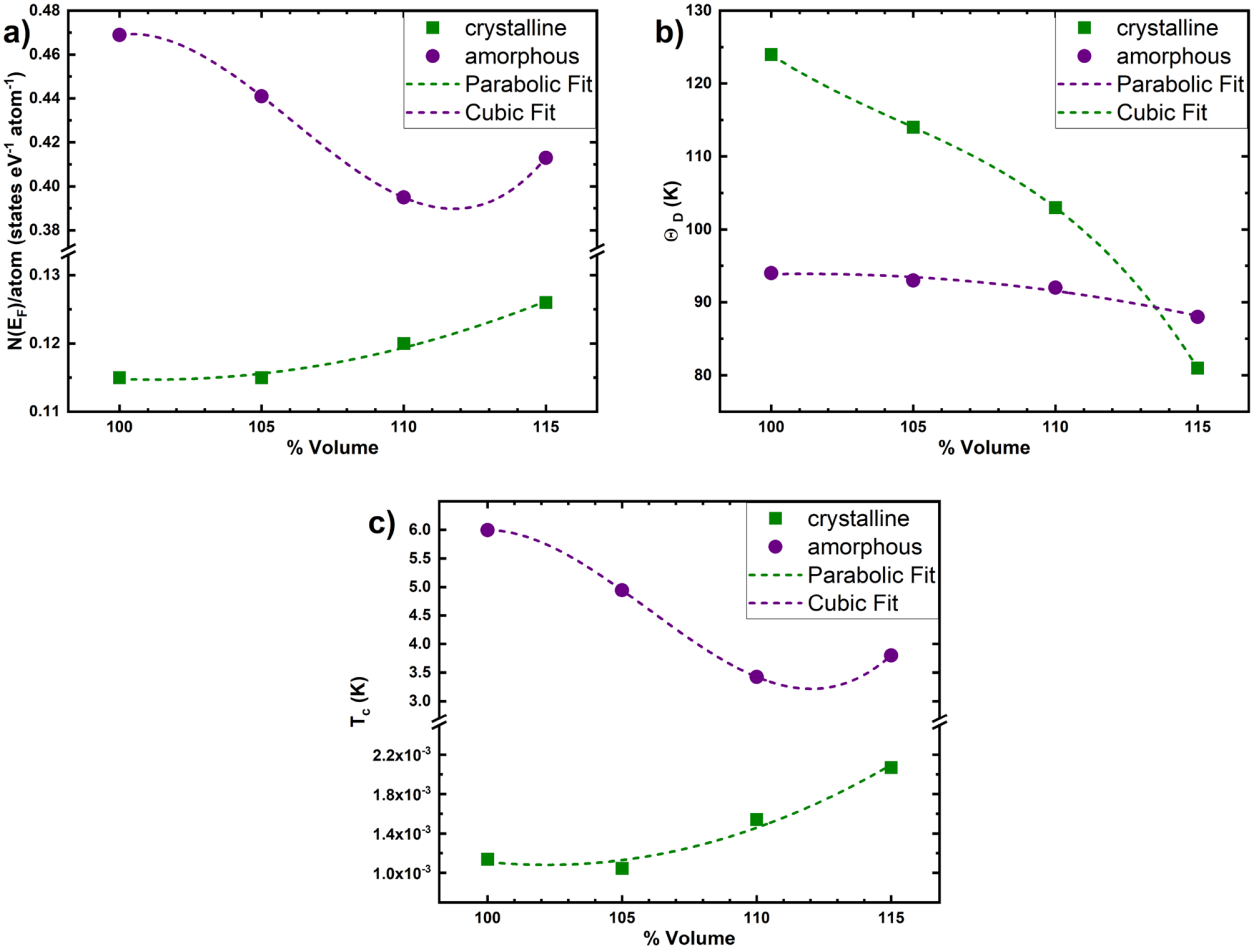
Graphical display of our results as a function of the expansion for crystalline and amorphous cells. Broken lines are parabolic and cubic fits for these results. (**a**) Electron densities of states (eDoS), (**b**) Debye temperatures (*θ*_*D*_), and (**c**) Superconducting transition temperatures (*T*_*c*_).

Figure 7 are a graphical representation of our results for a) *N*(*E*_*F*_), b) *θ*_*D*_, and c) *T*_*c*_ The general behavior is that the superconducting transition temperature varies minimally (from 1.14 to 1.05 mK, to 2.07 mK) for the crystalline structures as they expand, whereas it decreases notably (from 6.00 to 3.43 K to increase again) as the amorphous structures expand. It is surprising to see the similarity in the behavior of the eDoS at the Fermi level and the estimated *T*_*c*_ of the samples. On the other hand, the behavior of *θ*_*D*_ indicates that the value for the amorphous and for the crystalline materials decrease as they expand. Table 2 gives the numerical values for the graphs of Fig. 7.

**Table 2:** *N*(*E*_*F*_), *ϴ*_*D*_ and calculated *T*_*c*_ for the crystalline (c-) and amorphous (a-) samples studied.

System	N(E_F_) (states eV^−1^ atom^−1^)	ϴ_D_ (K)	T_c_ (K)
c-100%	0.115	124	1.14 × 10^–3^
c-105%	0.115	114	1.05 × 10^–3^
c-110%	0.120	103	1.54 × 10^–3^
c-115%	0.126	81	2.07 × 10^–3^
a-100%	0.469	94	6.00
a-105%	0.441	93	4.95
a-110%	0.395	92	3.43
a-115%	0.413	88	3.80

The values for the Debye temperatures of 124 K for the c-100% should be compared with 129 K calculated in Ref.^10^. Also, contrast 94 vs. 100 K^10^ for the a-100%. The value of *N(E*_*F*_*)* for the crystal diminishes due to the well-known existence of a pseudo gap at the Fermi level (Fig. 3).

## Conclusions

The effect of pressure on materials has been studied for a long time. However, most of the experimental work has been centered on applying pressures larger than the atmospheric. Here, we propose looking the other way; that is, applying pressures below the atmospheric in a systematic way to study materials. Some studies have been done along these lines, but the field is still open and deserves more attention.

In this work we confirm that upon amorphization bismuth goes from being a semimetal to a metal, increasing its density of electronic states at the Fermi level, and developing a soft phonon peak between 0 and 4 meV in the process. The consequences of such changes are that amorphous bismuth becomes a superconducting metal with a *T*_*c*_ (≈ 6 K) much higher than that determined for the crystalline phase, *T*_*c*_ ≈ 0.5 mK, and that we predicted to be less than 1.3 mK in 2016 (Ref.^10^). However, upon expansion amorphous bismuth becomes less metallic and the eDoS decreases, whereas the crystalline bismuth pseudo gap becomes less pronounced and the eDoS increases. The expansion herein considered may affect the physical properties of bulk Bi; in particular, we predict that the superconducting properties may be altered. Although the changes are not breathtaking from the industrial viewpoint, the search for a room temperature superconductor must go on.

No alteration of the structure of the crystalline cells upon expansion have been considered and since the Wyckoff structure is one that would tend to become cubic (look at the closeness of the two nearest-neighbor peaks in Fig. 1) it would be likely that any perturbation on the topology may induce rearrangements. Also, we have assumed that the electron-phonon interaction is the same for the amorphous and crystalline phases and that they can be described by the BCS formalism.

## Data Availability

The datasets analyzed during the current study are available from the corresponding author on reasonable request.

## Code availability

Materials Studio is a licensed software acquired from Dassault Systèmes BIOVIA.
